# The relationship between reward context and inhibitory control, does it depend on BMI, maladaptive eating, and negative affect?

**DOI:** 10.1186/s40359-021-00712-5

**Published:** 2022-01-04

**Authors:** Afework Tsegaye, Cuiling Guo, Gijsbert Stoet, Renata Cserjési, Gyöngyi Kökönyei, H. N. Alexander Logemann

**Affiliations:** 1 Doctoral School of Psychology, ELTE, Eötvös Loránd University, Budapest, Hungary; 2 Institute of Psychology, ELTE, Eötvös Loránd University, Budapest, Hungary; 3 Department of Psychology, University of Essex, Colchester, UK; 4 NAP2-SE Genetic Brain Imaging Research Group, Hungarian Academy of Sciences, Semmelweis University, Budapest, Hungary; 5 Department of Pharmacodynamics, Semmelweis University, Budapest, Hungary

**Keywords:** BMI, Inhibition, Eating-behavior, Rumination, Stress, Reward

## Abstract

**Background:**

Recent studies suggest that higher Body Mass Index (BMI) is associated with reduced inhibitory control in contexts of palatable food. However, due to limitations of previous studies, it remained the question whether this reduction is specific to food contexts, and whether it generalizes to other contexts of reward, such as money. This main question was addressed in the current study. In addition, we explored the effect of maladaptive eating and stress regarding inhibitory control across the contexts that differed in terms of reward.

**Methods:**

In total, 46 participants between 19 and 50 years old (39% males and 61% females) with an average BMI of 23.5 (SD = 3.9) participated. Participants filled out questionnaires and performed a go/no-go task (indexing inhibitory control) with three conditions (neutral, food, and money condition).

**Results:**

Relatively high (above median) BMI was associated with challenged inhibitory control in the food relative to the neutral context, but not in the money relative to neutral context. Explorative analyses suggested that maladaptive eating and stress were associated with reduced inhibitory control in the food context. Only rumination was associated with reduced inhibitory control in the money context.

**Conclusions:**

The effects of BMI, maladaptive eating behavior, and stress on inhibitory control were specific to the food context, and did not generalize to a non-intrinsic reward condition, operationalized with money pictures. Our results imply that (research on) interventions directed at improving inhibitory control in relation to overweight and obesity, should consider food-reward context.

## Background

Developing a more thorough understanding of the mechanism of inhibitory control in relation to weight is of clear importance, especially viewing the staggering global increase in prevalence of overweight and obesity [1]. Overweight is characterized by a Body Mass Index (BMI) exceeding 25 kg/m^2^, whereas obesity is characterized by a BMI exceeding 30 kg/m^2^ [1]. Certainly, the environment may be regarded as one of the predominant drivers of this increased prevalence [2]. However, as not everyone develops overweight or obesity in an obesogenic environment, there must be other factors that contribute to weight change [2]. One such plausible factor is inhibitory control, the ability to withhold a prepotent response [3]. Previous studies suggest a bidirectional causal relationship between inhibitory control and BMI, with poor inhibitory control predicting increased BMI [4, 5], and with increased BMI due to overconsumption of palatable food (food high in sugar/fat content) plausibly resulting in reduced inhibitory control [6]. In that vein, it is important to note that maladaptive eating patterns, i.e. emotional eating [7] and uncontrolled eating [8], as well as negative affect, i.e. stress [9] and rumination [10], especially promote overconsumption of palatable food, and in turn may negatively affect inhibitory control. However, in a previous review it was shown that there is not a consistent relationship between increased BMI and inhibitory control in neutral contexts [3], but results from a relatively recent study suggest that higher BMI is associated with a specific reduction of inhibitory control in a context of palatable food [11]. The open question is whether the relationship between higher BMI and challenged inhibitory control is specific to a context of palatable food, or whether it reflects a generalized reduction of inhibitory control in any reward context. This was the main question addressed in the current study. In addition we explored the relationship between maladaptive eating, negative affect on the one hand, and inhibitory control across reward-contexts on the other.

Though several studies have focused on the effect of inhibitory control on BMI, other studies suggest that increased BMI may predict challenged inhibitory control, suggesting a bidirectional causal relationship. Pertaining to the mechanism of the latter, normally, perceiving reward related stimuli (e.g. palatable food) results in increased striatal dopaminergic neurotransmission, and makes approach behavior more likely [12, 13]. Meanwhile, inhibitory control driven by dopamine D2 fronto-striatal circuitry [14], controls approach behavior to rewards [6]. However, chronic overexposure to rewards is thought to result in sensitization, increased attention and approach bias to rewards and associated stimuli together with downregulation of dopaminergic D2 and reduced dopamine D2 neurotransmission [6, 15, 16]. This is consistent with previous studies that show enhanced attentional bias for food related stimuli in adults with obesity [17]. In turn, the downregulation of dopamine D2 is thought to negatively affect fronto-striatal driven inhibitory control of approach bias for rewards [6]. This notion from the dopamine motive theory as postulated by Volkow et al., is compatible with Robinson’s [18] incentive sensitization (IS) theory. The latter theory posits that chronic exposure to rewards enhances sensitivity and associated attention for rewards after repeated exposure, via sensitization of the dopamine system, also termed “wanting system”. The inverse is evidenced for the “liking” system/response, which becomes less responsive over time [18, 19].

Studies that focused on behavioral indices of inhibitory control in relation to BMI are consistent with the above. Firstly, no consistent relationship between BMI and inhibitory control in neutral contexts is evidenced, but the relationship depends on both subject and environmental (reward context) variables [11]. With respect to the latter, a relatively recent study showed that higher BMI was associated with reduced inhibitory control, but the relationship was specific for the condition in which participants were shown pictures of palatable food (snacks). This is also supported by other studies. For instance, Batterink et al. [20] reported an inverse relationship between BMI and inhibitory responses to food-related stimuli in a go/no-go task. In addition, higher BMI is associated with poor inhibitory control especially to high-caloric food stimuli relative to low-caloric food stimuli [21].

One open question that has not yet been thoroughly addressed, is whether the challenged inhibitory control is specific to a food context, or whether it generalizes to other reward contexts as well. The latter may be expected following incentive sensitization theory which predicts that over time, sensitization to one type of reward(-related stimulus) extends to other reward(-related) stimuli as well through cross-sensitization [18, 19]. The known comorbidity between eating disorders associated with significant weight gain and substance use disorder provides some additional support for this notion [22].

With respect to eating behaviors, it should be emphasized that especially uncontrolled/binge eating [8] and emotional eating [7] has been shown to promote overconsumption of palatable food. Pertaining to the latter, rumination and stress may also play an important role. To elaborate, both stress and rumination have been associated with a shift in preference and overconsumption of palatable foods [9, 10] and rumination has been shown to be associated with increased BMI [23, 24]. This is perhaps not surprising as palatable food may serve as a potent negative reinforcer subsequent in a context of negative affect. Specifically, previous studies have shown that a state of negative affect is reduced subsequent to ingestion of palatable food [25].

It should be noted that stress may also have a moderating role regarding the relationship between BMI and inhibitory control, which may be most prevalent in a food context. In other words, the relationship between BMI and inhibitory control may vary as a function of stress. The study of Nederkoorn et al. [26] is noteworthy in that respect. In that study inhibitory performance was assessed with the Stop Signal Task in individuals with obesity and compared to those with normal BMI. Congruent with the above discussion, no main effect of group was evidenced with respect to inhibitory control, but group significantly interacted with time on task, with obesity associated reduced inhibitory control evident only in the last part of the task. As noted previously, the stop signal task is a relatively frustrating and stress inducing task [27], especially since the task is tailored to yield an approximate 50% failed inhibition rate [28]. This implies that the reduction in inhibitory control in obesity as time progressed, may be due to induced stress. In other words, the relationship between BMI and inhibitory control may vary as a function of stress.

In the current study, we measured inhibitory control using a go/no-go task modeled after Wessel [29], that included a neutral, food, and money condition. Though a stop signal task would also be suitable, the go/no-go task is easier to implement and no-go trials in the go/no-go task are also reported to trigger the inhibitory brain mechanism [29].

Integrating the above, it was hypothesized that higher BMI as well as related maladaptive eating patterns would be associated with reduced inhibitory control in reward contexts relative to a neutral context. In addition, the same was hypothesized with respect to rumination and stress, while stress was also hypothesized to moderate the relationship between BMI and inhibitory control, especially in a food context.

## Methods

### Participants

Participants were recruited predominantly via advertisements on social media (e.g. Facebook, LinkedIn). Participants were excluded if they had any known current mental disorder, if they currently used drugs affecting cognitive functioning, if they had color blindness and if they were pregnant. Participants had to be between 18 and 50 years old. Participants who did not complete the experiment were excluded. The final sample for final analyses consisted of in total forty-six participants (39% males and 61% females), ranging in age from 19 to 50 years old (M = 30.80, SD = 9.32) and Body mass index BMI (kg/m^2^), (M = 23.49, SD = 3.85). In total 34 participants had normal BMI (range 19 to 25, M = 21.75, SD = 1.80) with a gender distribution of 40% males and 60% females respectively, and 12 participants (BMI range 25 to 39, M = 28.42, SD = 3.92; equal gender distribution) presented with overweight (n = 8) or obesity (n = 4). The study was approved by the Research Ethics Committee and conducted following the declaration of Helsinki. Written informed consent was obtained from all participants.

## Materials

### Go/no-go task

**Fig. 1:**
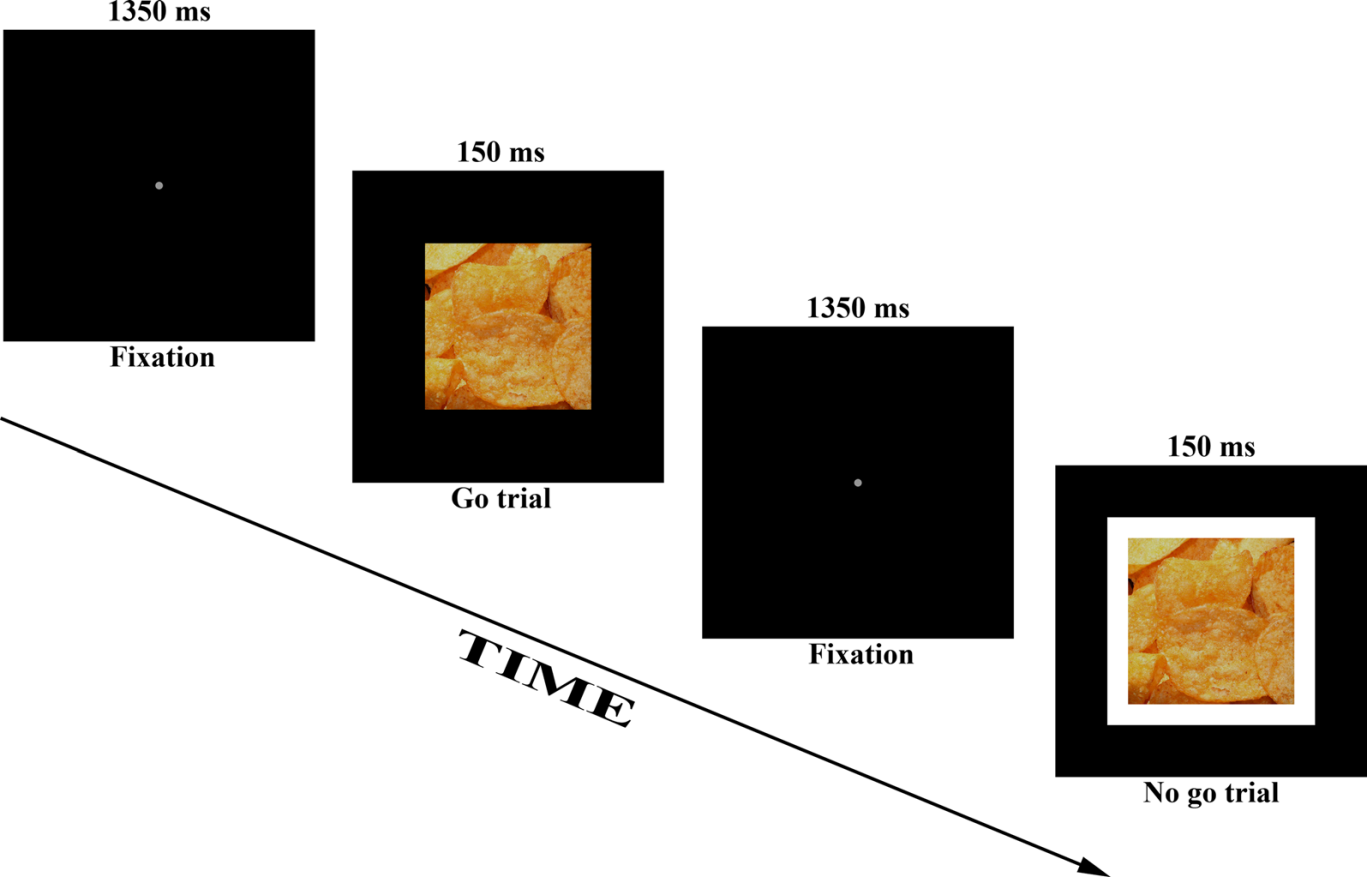
Schematic representation of two trials in the food condition of the Go/no-go task

The go/no-go task [29] was employed to assess inhibition. A single trial consisted of the presentation of a target stimulus, a central go stimulus (400 × 400 pixels) requiring a space-bar response or no-go stimulus (400 × 400 pixel go stimulus but surrounded a 50-pixel white border) to which no response should be made. The duration of the target stimulus was 150 ms and subsequently a fixation dot was presented until the next target stimulus was presented. The trial-to-trial duration was 1500 ms. Probability of a go stimulus was 80% and of a no-go stimulus 20%. The task consisted of one practice block and three experiment blocks (neutral, food and money condition). The practice block consisted of 8 trials and feedback was provided in case of an error of commission or error of omission. The three experimental blocks/conditions consisted of 40 trials. The main difference between the blocks were the pictures. In the practice block, the target stimulus was one of four possible gray squares. For the neutral, food and money condition the target stimulus was respectively one of four possible solid color filled squares (olive, green, blue, orange), pictures of food or pictures of money. The measure of inhibitory control was the proportion of successful inhibitions to no-go trials (number of successful inhibitions to no-go trials divided by the total number of no go-trials) (Fig. 1).

## Self-report measures

### Three-Factor Eating Questionnaire-R18V2 [30]

The participants eating behavior was assessed by the short version of the three-factor eating questionnaire (TFEQ-R18). Previous research has shown that the TFEQ-R18 yields a good measure of uncontrolled and emotional eating and has a good factor structure and reliability with Cronbach’s alpha ranging between 0.78 and 0.94 [30]. The TFEQ-R18 has three subscales: cognitive restraint of eating (conscious restriction of food intake in order to control body weight, three items), uncontrolled eating (tendency to eat more than usual due to a loss of control, nine items) and emotional eating (inability to resist emotional cues/ responding to negative emotions by eating, six items), in total 18 items. Participants rate each item to the extent that the item-content applies to his or her thoughts, feelings and behaviors on a scale of 1 to 4, scoring 1 equal to "definitely false" and scoring 4 equal to ‘definitely true.’ The range of possible scores for each subscale is 9 to 36, 6 to 24, and 3 to 12 points for the uncontrolled eating, emotional eating scale, and cognitive restraint respectively. Higher scaled scores for each subscale suggest greater UE, CR or EE characteristics the internal consistency of subscale showed good reliability (Cronbach’s alpha: 0.89, 0.78 and 0.94, respectively) [30]. Cronbach’s alpha in our study 0.776, 0.733 and 0.959.

### Ruminative response scale (RRS) [31]

The RRS is one of the most popular instruments to assess ruminative responses [31], and has excellent reliability with a Cronbach’s alpha across studies of about 0.9. The RRS is a 22-item scale capturing trait-like ruminative thoughts *(“What am I doing to deserve this?”)* when experiencing low mood. Items are answered on a 4-point Likert-type scale, ranging from 1 "almost never" to 4 "almost always"; higher scores reflect higher rumination. In our study Cronbach’s alpha was 0.943.

### Depression, anxiety and stress scale (DASS-21) [32]

The DASS-21 is a widely used instrument to assess levels of depression, anxiety and stress by self-report, also in relation to clinical symptomatology. The instrument has a good overall reliability with a Cronbach’s alpha of 0.91, 0.84 and 0.80 for the Depression, Stress and Anxiety subscale, respectively [33]. Each of the three subscales contains 7 items. Items are answered on a 4-point Likert-type scale, ranging from 0 "Never" to 3 "Almost always". Scores for stress, anxiety and depression scales are determined by summing the scores for the relevant 7 items and multiplying by 2. For the current study, we only focused on the stress scale and Cronbach’s alpha for this scale was 0.867.

## Procedure

The assessments were completed online, via Psytoolkit [34, 35]. All participants were provided with basic information regarding the study and procedures and read and provided their written informed consent After providing informed consent, the experiment started with self- report general questions that included question regarding age, gender, weight, height, smoking status, handedness, hunger level and amount of sleep last night. Subsequently, participants filled out psychometric scales. Finally, participants performed the go/no-go task. During this task, pictures were presented sequentially, and participants were required to respond via pressing the spacebar unless the picture was surrounded by a white border. In the latter case, a response was required to be withheld. Upon completion of the go/no-go task, the study was concluded. The total duration of the study (questionnaires and experiment) was approximately 25 min.

### Statistical approach

Repeated measures ANCOVAs were performed for the primary analyses, with alpha level set at 0.05. Analyses were similar to the ones performed in Houben et al. [11], and one of the aims was to test whether their results were replicable. Specifically, we tested the BMI × condition (neutral/food/money) interaction regarding the proportion of inhibitions to no-go trials. As the relationship between BMI and inhibitory control does not necessarily follow a linear relationship, as described in Houben et al. [11], we also included BMI as categorical variable in the model (repeated measures ANOVA) as a secondary approach. Specifically, to maintain equal sample sizes we transformed BMI to a categorical variable via median-split, creating two levels (below median/above median). Lastly, we tested the interactions between maladaptive eating (uncontrolled and emotional eating), rumination and stress on the one hand and condition (neutral/food and neutral/money) on the other.

## Results

Descriptive data for age, BMI, eating behavior, rumination, stress and subjective hunger level is shown in Table 1. Participants that had 40 percent or more omissions in go trials in the GNG task were excluded from data analyses, see Table 2 for the proportion of omission and inhibitions for the final sample (N = 46).

**Table 1:** Descriptive data eating behavior, rumination, stress, age, BMI, and subjective hunger level (N = 46)

	Scale-range (max–min)	Max (obtained)	Min (obtained)	M (mean)	SD (std. deviation)
Age	50–18	50	19	30.8	9.3
BMI	N/A	39	19	23.5	3.9
Stress	42–0	28	0	9	7
Rumination	88–22	73	23	42	12
Hunger level	5–1	4	1	2	1
Uncontrolled eating	36–9	33	11	19	5
Cognitive restraint	12–3	12	3	6	3
Emotional eating	24–6	21	6	11	4

**Table 2:** Omissions and inhibitions in the three conditions of the GNG task (N = 46)

Condition	Variable	Maximum	Minimum	Mean	SD
Neutral	Omissions	0.28	0	0.02	0.06
Food	Omissions	0.19	0	0.02	0.04
Money	Omissions	0.13	0	0.01	0.02
Neutral	Inhibitions	1	0	0.77	0.23
Food	Inhibitions	1	0	0.73	0.23
Money	Inhibitions	1	0.25	0.73	0.19

Omissions: proportion of no-response in go-trials; inhibitions: proportion of correct inhibitions in no-go trials

### Primary analyses: BMI and inhibitory control across different conditions

**Fig. 2:**
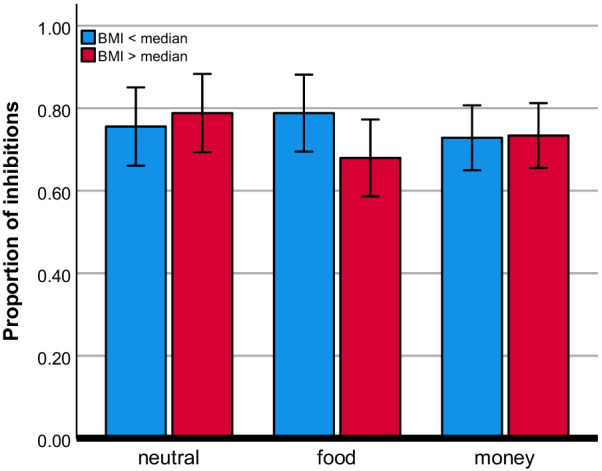
Mean proportion of inhibitions for both BMI groups across the three conditions, neutral, food, and money. Error bars indicate ± 2 standard errors from the mean

The relationship between BMI and inhibitory control did not significantly vary as a function of condition (neutral/food/money), F(2,88) = 0.48, *p* = 0.622, partial η^2^ = 0.011. The secondary analysis, including BMI as categorical variable based on split by median (= 22.43) yielded a BMI (above median/below median) × condition (neutral/food/money) interaction that trended to significance, F(2,88) = 2.73, *p* = 0.071, partial η^2^ = 0.058. Post-hoc analyses indicate that above median BMI relative to below median BMI was associated with reduced inhibitory control in the food condition relative to the neutral condition, F(1,44) = 4.12, *p* = 0.049, partial η^2^ = 0.086 (see Fig. 2). This relation was not evident for the neutral/money contrast, F(1,44) = 0.20, *p* = 0.660, partial η^2^ = 0.004. Lastly, there was no main effect of condition regarding inhibitory control, F(2,88) = 1.01, *p* = 0.369, partial η^2^ = 0.022.

### Secondary analyses: maladaptive eating behavior, rumination, stress and inhibitory control across the different conditions

As shown in Table 3, there were no significant main effects or interactions with condition with respect to eating behaviors, rumination and stress regarding inhibitory control. Explorative correlational analyses indicated that maladaptive eating, rumination and stress were associated with reduced inhibitory control, specifically in the food context. Only rumination was negatively associated with inhibitory control in the money context (Table 4).

**Table 3:** Inferential statistics regarding eating behavior, rumination and stress with respect to inhibitory control

Factor	F	*p*	Partial η^2^
Uncontrolled eating × condition (neutral/food/money)	F(2,86), 1.00	0.372	0.023
Uncontrolled eating	F(1,43), 0.06	0.807	0.001
Emotional eating × condition (neutral/food/money)	F(2,86), 1,55	0.219	0.035
Emotional eating	F(1,43), 0.16	0.695	0.004
Rumination × condition (neutral/food/money)	F(2,86), 0.19	0.83	0.004
Rumination	F(1,43), 1.22	0.276	0.001
Stress × BMI × condition (neutral/food/money)	F(2,84), 1.41	0.251	0.032
Stress × condition (neutral/food/money)	F(2,84), 1.88	0.159	0.043
Stress	F(1,42), 0.70	0.409	0.016

**Table 4:** Post-hoc explorative correlational analyses, regarding the relationship between self-report measures and inhibitory control in the neutral, food, and money condition (n = 46)

	Neutral	Food	Money
Uncontrolled eating	0.065	− 0.248^a^	− 0.026
Emotional eating	0.013	− 0.319*	− 0.167
Rumination	− 0.147	− 0.390**	− 0.299*
Stress	− 0.038	− 0.323*	− 0.053

*Correlation is significant at 0.05 level (2-tailed); **Correlation is significant at 0.01 level (2-tailed); ^a^Trending to significance, *p* < 0.1

## Discussion

Our results indicate that higher BMI is associated with reduced inhibitory control in a food context relative to a neutral one. In addition, explorative correlational analyses seem to indicate that maladaptive eating and negative effect were associated with reduced inhibitory control in the food context. In contrast to our hypotheses, effects with the exception of rumination, did not generalize to the money context.

In a previous study, it was already reported that higher BMI was associated with reduced inhibitory control in a food context relative to a neutral context. However, one important limitation challenged the interpretation of effects. More specifically, in Houben et al. [11] counterbalancing of conditions was omitted, meaning that all participants were provided with the same order of tasks, neutral condition first followed by the food condition. This means that the interaction between BMI and condition regarding inhibitory performance, could also be explained by an interaction between BMI and time. Importantly, a previous study from Nederkoorn et al. [26], in which only a neutral stop task was employed, reduced inhibitory control in the group with obesity relative to the normal weight group, was only evident at the last part of the task. This means, that the effect reported in Houben et al. [11], could potentially be due to time (and potentially induced stress) instead of reward context. This limitation was addressed in the current study in which counterbalancing of condition order was employed. As mentioned, results are congruent with previous findings [11, 20].

In line with previous reports [36], the explorative correlational analyses suggest that maladaptive eating patterns that promote overconsumption of palatable food (uncontrolled eating and emotional eating) are associated with challenged inhibitory control, specifically in a food context. This is an important finding as, to the best of our knowledge, previous studies have not thoroughly assessed food specific inhibitory effects, as most studies omitted a control condition that included non-reward stimuli. As hypothesized, rumination was also associated with reduced inhibitory control in both reward contexts. This finding supports the notion that trait rumination may affect reward-related information processing. Interestingly, in a previous report, using the monetary incentive delay (MID) task in a never-depressed sample a significant positive association was found between trait rumination and neural response in areas of the Salience Network to monetary reward anticipation (reward cues) compared to loss anticipation (loss cues) [37]. However, the MID task differs from the go/no-go task used in our study in many ways, both studies show that reward processing might be altered in relation to rumination. Further studies need to investigate whether the relationship between rumination and reward processing may vary as a function of the type of the reward or the process (e.g. inhibition, switching, anticipation etc.) implemented in task performance.

As mentioned, it was expected that the negative association between BMI, maladaptive eating, stress and rumination on the one hand and inhibitory control on the other in the food context relative to the neutral context, would be mirrored in the contrast between the money context relative to neutral context. However, except regarding rumination, our results did not support that notion. One might argue that this implies that the challenged inhibitory control is restricted to food contexts and may not generalize to other reward contexts. However, one limitation of the current study may be the operationalization of the non-food, reward condition operationalized by stimuli representing money. We employed the money condition because stimuli of money are obviously different than food, but are also known to trigger primary reward related activity [38]. However, it should be noted that money not only represents a different reward object, but also a different class of reward. To elaborate, whereas food can be regarded as an intrinsic reward, money related stimuli become rewarding/reinforcing only after an operant learning process over time. Now as mentioned, the mechanism implicated in obesity shows significant overlap with that implicated in substance use disorder [39], and cross sensitization for different substances and associated stimuli may (at least partly) explain comorbidity between maladaptive eating and substance use disorder. However, following our results, one might argue that cross sensitization is more restricted to reward related stimuli that are intrinsically rewarding as opposed to non-intrinsic reward related stimuli. The latter would be one question that could be addressed in future studies.

On the other hand, results from Godefroy et al. [40], may suggest an alternative explanation. They employed a structural equation modeling approach and showed that effortful control was associated with enhanced self-regulation in eating, which in turn, was associated with reduced appetite reactivity. This may suggest that, even though cross-sensitization may enhance motivational and response tendencies towards other reward-associated stimuli (in line with e.g. Tsegaye et al. [12, 13], maladaptive eating and associated adiposity due to reduced temperamental inhibitory control, may be specifically evident in a palatable food context.

It should be noted that the relation between BMI and inhibitory control in the food context relative to the neutral context was only statistically significant in our exploratory analysis when including BMI as a categorical (median split) variable. Following Houben et al. [11], we decided to perform such approach as the relationship between BMI and inhibitory control is not necessarily linear. One may note that one disadvantage of a median split strategy is that it can make results relatively sample dependent, as groups are not formed based on an apriori criterion regarding group assignment. For instance, the latter would entail assigning participations to an overweight/obesity group and control group based on BMI value. However, in the current study the aim was not to specifically focus on overweight and/or obesity, but to assess the general relationship between BMI and inhibitory control. Importantly, testing the difference between the overweight/obese group and normal healthy weight controls would result in unequal sample sizes and especially small sample size of the overweight/obese group.

One other limitation, at least perceptually, is that the study was conducted online. However, it should be emphasized that recent studies consistently show that online cognitive psychological experiments can generate reliable and valid data, comparable to those conducted in a controlled lab environment [41, 42]. Of course, it is still possible that participants did not take the experiment seriously or did not understand the task. However, we controlled for this potential issue and excluded those participants that did not respond consistently to go trials, indicated by the proportion of omissions to those trials.

Furthermore, the use of the go/no-go task as opposed to the stop signal task, to measure inhibitory control, could be viewed as a limitation. Firstly, there is substantial heterogeneity of go/no-go task implementations in terms of task specifics. Until recently, it was not entirely clear what task characteristics were essential to induce inhibitory control. However, as shown in Wessel et al. [29], a fast-pace go/no-go task (with 1500 ms intertrial interval), with infrequent (20%) no-go trials triggers inhibitory control, comparable to that commonly reported in stop signal paradigms. In that vein, as our go/no-go task used these same task characteristics, the relevant outcome variable (proportion of inhibitions) is thought to reflect inhibitory performance.

## Conclusion

Our results indicate that higher BMI, maladaptive eating patterns (uncontrolled eating and emotional eating) as well as rumination are associated with challenged inhibitory control when perceiving palatable food related stimuli relative to neutral stimuli. We could not confirm that the effect generalizes to a different, and non-intrinsic reward context operationalized by money. The results have clinical implications in that interventions aimed at improving inhibitory control may benefit from a focus on inhibitory control in a context of palatable food. Specifically, it has been shown that strengthening inhibitory control can assist individuals with obesity in regaining control over the consumption of palatable foods, as well as making them less susceptible to the temptations of palatable food [43, 44]. Our results underscore the importance of food specific inhibitory training as one of the promising intervention methods for changing bad eating habits and losing weight.

## Data Availability

The materials, data, and data-processing scripts are available in a public repository, at the Open Science Framework (osf.io; https://doi.org/10.17605/OSF.IO/2TFWZ).

## Abbreviations

ANOVA: Analysis of Variance
ANCOVA: Analysis of Covariance
BMI: Body Mass Index
DASS: Depression Anxiety Stress Scale
GNG: Go/No-Go
IS: Incentive Sensitization
M: Mean
MAX: Maximum
MID: Monetary Incentive Delay
MIN: Minimum
RRS: Ruminative Response Scale
SD: Standard Deviation
TFEQ: Three-Factor Eating Questionnaire
WHO: World Health Organization
